# A Critical Clinical Study of Hedonal

**Published:** 1903-10

**Authors:** A. Toepfer

**Affiliations:** Jersey City, N. J.


### A Critical Clinical Study of Hedonal.

BY A. TOEPFER, M. D., Jersey City, N. J.

IT IS a matter of common observation that while our resources for combating comparatively trivial diseases are quite meagre, this assertion does not hold good for the more serious maladies. What physician has not been impressed with the inadequacy of our medical knowledge in the case of toothache, or coryza, or lumbago, or dysmenorrhea, and the like, although his skill often succeeds in bringing to a favorable termination the dangerous inflammatory diseases? How often is not the physician compelled to give evasive answers when requested to relieve insomnia; for while the materia medica contains drugs which will produce narcosis, sopor, and coma, they are far from being capable of causing natural physiological sleep. For this reason when my attention was drawn to a number of reports from prominent European clinicians on the value of hedonal in conditions of simple sleeplessness, I was led to give it a trial in order to determine how far these laudatory statements were justified by the results of my own experience.

Hedonal is a derivative of the urethane group, and appears in the form of a crystalline white powder, almost insoluble in cold water, but easily dissolving in hot water and alcohol. It has a somewhat disagreeable, minty taste, which is accentuated when it is administered in water. In spite of this fact, however, it is well tolerated by the stomach, and during continued use any existing aversion soon disappears.

The best form of administration would undoubtedly be in tablets coated with chocolate, but as these are not obtainable in America I have given it in capsules, which, though less convenient, have proved satisfactory. I have also administered it in wafers, although some of the patients objected to their size. The suggestion to prescribe hedonal in a mixture with brandy and syrup is less applicable than the simple administration in an emulsion in water with gum arabic, because hedonal sometimes has a diuretic effect, thus disturbing sleep, and as the addition of alcohol, owing to its stimulating effect upon the heart, has a similar action, their conjoint administration might increase the chance of this taking place. It is further advisable to give no fluids for several hours before the administration of hedonal, so that the quantity of urine eliminated is diminished as much as possible. This diuretic action was observed by me more frequently in men than in women. I also noticed that if this effect manifested itself at all it persisted during each subsequent administration. I met with but few cases, however, in which the desire to urinate robbed the patient of further sleep, the majority promptly falling asleep again. On the other hand, the observation that the hypnotic action of hedonal is impaired by the previous ingestion of a hearty meal is based upon actual facts. According to my experience, at least three hours should be allowed to elapse before an ordinary meal and the administration of hedonal. If the patient, however, take a light repast or a little fruit, it does not interfere with the effect of the drug.

Another peculiarity in the action of hedonal was observed by me in a few instances,—namely, if the patient for some reason or other had taken a dose of bicarbonate of soda and hedonal was administered shortly thereafter, its hypnotic effect was less decided. If, however, some acid remedy was taken the hypnotic effect was strongly manifested.

Whether the sleep produced is dreamless, as maintained by some, I was unable to determine. I can positively state in regard to myself that after taking hedonal I woke up dreaming, as is my custom. What is of more importance, however, is the question as to the conditions necessary for the occurrence of sleep. Hedonal is not an absolute hypnotic which manifests the same effect at all times and in all persons, its action depending upon certain requirements. Just as normal sleep implies a condition of restfulness of the central nervous system, there must be present a desire for rest and sleep if hedonal is to act. If this is wanting, whether in consequence of marked psychical excitement, especially if it be due to anxiety or fear, or the result of alcoholic excesses, the hypnotic effect frequently fails to manifest itself. On the other hand, if the sleeplessness is caused simply by mental depression or mental strain without the mind being concentrated upon a concrete subject, the effect leaves nothing to be desired. It is true that two cases of this kind came under my observation in which hedonal failed to act even in increased doses, but I could notice no difference between these and the other cases, since no objectively demonstrable reason for the inequality of its action could be discovered.

In the presence of pain I have not employed hedonal as a hypnotic, having always found aspirin a sovereign remedy for this purpose. If, however, after the removal of the pain the sleeplessness persists a dose of hedonal proves a valuable auxiliary. Whether experience will confirm my observations that in conditions of psychical excitement the mental equilibrium necessary for the occurrence of sleep is restored by a previous dose of aspirin, I am unable to decide positively. Sleep usually occurred one-half to one hour after the administration of the drug, the most rapid results being obtained from the larger doses, as I had occasion to frequently observe. In regard to its character, the sleep cannot be distinguished from the normal slumber, which is in itself shown by the facts, that the patient assumes an easy position, that the respiration is regular and not stertorous, and the pulse remains normal. For the latter reason the diuresis, which occasionally occurs, cannot be attributed to an increased pressure in the renal capillaries; it must be a direct consequence of the irritation of the epithelia of the glomeruli. As to the duration of the sleep produced by hedonal no exact data can be given. In the cases observed by me the time of continued sleep varied from two to five hours, the patients usually falling asleep again.

One factor which distinguishes hedonal from all other hypnotics is its property of leaving the physical and mental powers unimpaired. It is true that in making this statement I rely upon tests made upon myself, since confirmatory control experiments were not for obvious reasons available. It has happened that within a short time, only two or three hours after taking a dose of hedonal at night, I was awakened by a call, and found myself mentally and bodily as active as one is accustomed to be when aroused from natural sleep. This freedom of the drug from any influence upon the mental and bodily powers I have failed to note in any other hypnotic, although I have tested the entire list; for since my fiftieth year I have often experienced difficulty in falling asleep for a number of hours after having been disturbed at night. At the present time I take refuge in hedonal in that event. Another advantage of hedonal which ought not to be underestimated is its property of not interfering with the pulse, a quality which is lacking in all other hypnotics.

Unpleasant by-effects, such as eructations or vomiting, have been observed in a slight degree in a few instances. A slight feeling of vertigo, easily controlled, followed occasionally after large doses soon after its administration, sometimes while urinating. Some persons taking the drug for long periods remarked during the day a transient itching in various parts of the body, usually the scalp. Injurious effects were never noted. According to the literature published, a few authors have observed a weakening of the hypnotic influence after the prolonged administration of the remedy. My control experiments lead me to a contrary opinion, although I have always resorted to medium-sized doses. Cumulative effects seem to be absent with hedonal.

In regard to the indications I have always confined myself within the narrow limits of insomnia unattended with pain, without, however, neglecting to ascertain what special conditions of sleeplessness are most favorably influenced. Unfortunately, I must confess that my endeavors in this respect have not afforded me any special data, since the majority of my patients suffered with simple sleeplessness in consequence of advanced age, while the others comprised hysterical, neurasthenic, gouty, diabetic, melancholic, and alcoholic persons. The effect in children and young persons could not be determined, the age of the youngest patient being 35 years. The dose is not fixed, one is relieved by a small dose, and another by a large one. In all cases I commenced with 15 grain doses, and if the effect was satisfactory this was continued. The administration of two successive doses in the evening seemed to me unsuitable for determining the individual dose. If the first dose failed to afford the desired effect, then I increased it by one-half on the following night. If this dose (22 grains) did not induce several hours sleep I added another seven grains on the following night, but never exceeded one-half drachm, although in the literature cases are reported in which far larger doses were given without any ill-effects. Fifteen grains were sufficient only in the minority of cases, mostly women, without the necessity of increasing the dose. In the majority 22 grains were required, while 30 grains were necessary in a number of cases of neurasthenia with marked nervous excitement.

The following cases will serve to illustrate what has been said above:

Case I.—Mrs. R., 40 years old, member of a strongly neuropathic family, extremely eccentric. She presented aside from occasional sleeplessness no objective signs of disease. Her usual condition of sleep is as follows: she falls asleep at once on retiring, but wakes up after one or two hours and remains sleepless for the rest of the night. She was ordered to take hedonal, 15 grains, in two gelatin capsules as soon as she awoke, with the following result: she slept uninterruptedly until six o'clock in the morning, sleep ensuing one-half hour after taking the drug. She felt much invigorated and did not experience the slightest inconvenience.

Case II.—Mrs. H., 38 years old, a well-built blonde of hysterical tendency; married twice, her second husand being a young man. In consequence of this she was extremely jealous. In the presence of strange women the slightest inattention on the part of her husband was always followed by attacks of weeping and sleeplessness. Being consulted on one of these occasions I administered hedonal, 15 grains, with the result that three-quarters of an hour after its ingestion a quiet sound sleep ensued. She was compelled, however, to get up to urinate, but immediately fell asleep again. She now takes the remedy whenever required.

Case III.—Mr. S., a robust man, 50 years old, subject to going on sprees, but a total abstainer during the intervals, frequently suffers from insomnia and restlessness at night. The first dose of hedonal, 15 grains, had no effect. On the following night he received 22 grains, and an hour later he became somnolent and failed to hear the striking of the clock, but only once, for after that he heard it each time. On the third night I prescribed 30 grains of hedonal, and this was found to be the appropriate dose for producing quiet invigorating sleep until morning. If later he reduced the dose the result was always a failure.

Case IV.—I, myself, experimented with hedonal for several weeks in order to test its action upon the psychical and intellectual faculties. One evening about 10 o'clock, I was called to a patient who had suffered for two hours from violent nose-bleed. At midnight I finally succeeded in stopping the hemorrhage. Being exhausted and having been deprived of my sleep, I took 22 grains of hedonal before retiring. Sleep must have occurred at once, since I cannot remember having heard the clock strike. About two o'clock, however, my night bell rang, and my first thought was of the old gentleman and of the possibility of a recurrence of the epistaxis, but fortunately it was a different affair. A man desired a remedy for relieving his wife of a violent attack of colic. Notwithstanding that when I was aroused I was in the first stage of hedonal sleep I experienced neither dizziness nor a dulling of the senses, so that I was perfectly able to think over the case and dispense the proper remedy. A severer test in regard to the complete innocuousness of hedonal in reference to the mental and bodily power could not be readily found.

I would formulate my conclusions based upon my observations (25 cases) as follows: in all uncomplicated cases of sleeplessness hedonal is a quite reliable and absolutely harmless hypnotic, this being due to the fact that it is oxidized in the organism into carbonic acid, urea, and water, and is free from any poisonous decomposition products. Even when employed for a long time its effect is not impaired and it promises to become a real blessing to the many sufferers from insomnia.

283 Central Avenue.

